# Reprogramming Intestinal Epithelial Cell Polarity by Interleukin-22

**DOI:** 10.3389/fmed.2021.656047

**Published:** 2021-04-12

**Authors:** Deborah Delbue, Lydia Lebenheim, Danielle Cardoso-Silva, Violaine Dony, Susanne M. Krug, Jan F. Richter, Subhakankha Manna, Melba Muñoz, Kerstin Wolk, Claudia Heldt, Markus M. Heimesaat, Robert Sabat, Britta Siegmund, Michael Schumann

**Affiliations:** ^1^Department for Gastroenterology, Rheumatology and Infectious Diseases, Charité—Universitätsmedizin Berlin, Corporate Member of Freie Universität Berlin, Humboldt-Universität zu Berlin and Berlin Institute of Health, Berlin, Germany; ^2^Institute of Clinical Physiology, Charité—Universitätsmedizin Berlin, Corporate Member of Freie Universität Berlin, Humboldt-Universität zu Berlin and Berlin Institute of Health, Berlin, Germany; ^3^Institute for Anatomy II, University of Jena, Jena, Germany; ^4^Department of Microbiology, Charité—Universitätsmedizin Berlin, Corporate Member of Freie Universität Berlin, Humboldt-Universität zu Berlin, Berlin, Germany; ^5^Department for Dermatology, Charité—Universitätsmedizin Berlin, Corporate Member of Freie Universität Berlin, Humboldt-Universität zu Berlin and Berlin Institute of Health, Berlin, Germany; ^6^Department of Dermatology, Venereology and Allergology, Psoriasis Research and Treatment Center, Institute of Medical Immunology, Charité—Universitätsmedizin Berlin, Corporate Member of Freie Universität Berlin, Humboldt-Universität zu Berlin, Berlin, Germany

**Keywords:** intestinal epithelial cells, barrier function, cell polarity, IL-22, tight junctions, MAPK, stat3

## Abstract

**Background:** Interleukin-22 (IL-22) impacts the integrity of intestinal epithelia and has been associated with the development of colitis-associated cancer and inflammatory bowel diseases (IBD). Previous data suggest that IL-22 protects the mucosal barrier and promotes wound healing and barrier defect. We hypothesized, that IL-22 modulates cell polarity of intestinal epithelial cells (IECs) acting on tight junction assembly. The aim of the study was to investigate IL-22-dependent mechanisms in the reprogramming of intestinal epithelia.

**Methods:** IECs were exposed to IL-22 at various concentrations. IECs in Matrigel® were grown to 3-dimensional cysts in the presence or absence of IL-22 and morphology and expression of polarity proteins were analyzed by confocal microscopy. Epithelial cell barrier (TER and sandwich assay) and TJ assembly analysis (calcium-switch assay) were performed. TJ and cell polarity protein expression were assessed by western blotting and confocal microscopy. Cell migration and invasion assays were performed. Induction of epithelial-mesenchymal transition (EMT) was assessed by RT-qPCR analysis and western blotting. Signaling pathway analyses were performed by phosphoblotting and functional assays after blocking STAT3 and ERK signaling pathways. Using the toxoplasma-model of terminal ileitis, IL-22-knock-out mice were compared to wild-type littermates, analyzed for barrier function using one-path-impedance-analysis and macromolecular flux (H3-mannitol, Ussing-chambers).

**Results:** IECs exhibited a barrier defect after IL-22 exposure. TJ protein distribution and expression were severely impaired. Delayed recovery in the calcium-switch assay was observed suggesting a defect in TJ assembly. Analyzing the 3D-cyst model, IL-22 induced multi-lumen and aberrant cysts, and altered the localization of cell polarity proteins. Cell migration and invasion was caused by IL-22 as well as induction of EMT. Interestingly, only inhibition of the MAPK pathway, rescued the TJal barrier defect, while blocking STAT3 was relevant for cell survival. In addition, ileal mucosa of IL-22 deficient mice was protected from the barrier defect seen in Toxoplasma gondii-induced ileitis in wild type mice shown by significantly higher Re values and correspondingly lower macromolecule fluxes.

**Conclusion:** IL-22 impairs intestinal epithelial cell barrier by inducing EMT, causing defects in epithelial cell polarity and increasing cell motility and cell invasion. IL-22 modulates TJ protein expression and mediates tight junctional (TJal) barrier defects via ERK pathway.

## Introduction

Interleukin-22 (IL-22) is a member of the interleukin-10 cytokine family that is primarily acting on epithelial cells, which is secondary to the expressional restriction of the IL-22-receptor-1 chain to epithelia (1–3). IL-22 is upregulated in inflammatory bowel diseases (IBD) as Crohn's disease but also in coeliac disease (4–6). Although there are many reports on IL-22-mediated effects on epithelial cells, the overall outcomes described so far appear to be very heterogeneous. Dependent on the cell type involved or the type of inflammatory trigger, IL-22 was reported to result either in protection of epithelia/wound healing or to induce epithelial damage (1, 7). Specifically, IL-22 protected from colitis in infectious or chronic inflammatory models or induced ileitis in the *Toxoplasma gondii* model (8–12). However, previous analyses of epithelial cell responses to IL-22 are mostly limited to wound closure assays or examination of epithelial proliferation and apoptosis (7).

Epithelial polarity describes a cellular program ensuring proper localization of distinct polarity-relevant molecular constituents (i.e., phospholipids and proteins) to the apical or the basolateral epithelial compartments as well as the coordinated assembly of intercellular junctional structures, including tight junctions (TJ) and adherens junctions (13). It is regulated through a complex network of proteins that are strongly conserved throughout evolution and is described to be dysregulated in inflammation and carcinogenesis (14). However, it is unknown how epithelial polarity and barrier function are regulated during chronic inflammation of the gut. Previously, dysregulation of the polarity protein Pard3 was found in celiac disease and was connected to celiac epithelial barrier defects (15).

Furthermore, the proinflammatory cytokines, such as TNF-α and interferon-γ, have been shown to disturb regular lumen formation in intestinal epithelial cysts as well as alter the epithelial polarity and barrier function (16). In this context, activation of the IL-22 receptor triggers signaling via various pathways including STAT3, AKT and MAPK/ERK, that are crucial for cell survival, proliferation, barrier integrity and establishment of cell polarity (17–20). Therefore, we hypothesized that IL-22 exposure directly modulates the epithelial apical complex and also the establishment of cell polarity, thereby regulating the barrier function.

In this study, we show that IL-22 impairs the intestinal cell barrier integrity by inducing a complex reprogramming of intestinal epithelial cell functions. Within this regulation, IL-22 induces EMT, modulates TJ, and polarity protein expression and mediates TJal barrier defects via ERK- but not STAT3- or AKT-pathway.

## Materials and Methods

### Cell Culture and TER Measurement

Caco-2, HT29/B6, and T84 were maintained in Minimum Essential Medium Eagle's (MEM, Gibco/Thermo Fisher, Waltham, MA, USA), RPMI-1640 and DMEM/Ham's F-12 (Corning, Wiesbaden, Germany) supplemented with 10% fetal bovine serum (FBS, Gibco/Thermo Fisher), 1% penicillin and streptomycin (Corning), respectively. IECs were seeded on PCF filters (0.4 μm; 0.6 cm^2^, Merck Millipore, Darmstadt, Germany) and grown to confluence for 7, 10 and 12 to 14 days in culture at 37°C in a 5% CO_2_ environment, respectively. IL-22 (Biolegend, San Diego, CA, USA) was added to both, the apical and basolateral compartments of transwell filters for times indicated and transepithelial resistance (TER) was measured using chopstick electrodes. The IEC filters were basolaterally exposed to additional proinflammatory cytokines as TNF-α (1,000 U/ml); IFN-γ (100 U/ml); IL-13 (10 ng/ml); TGF-β1 (20 ng/ml). These cytokines were from Prepotech (Hamburg, Germany).

### Impedance Spectroscopy

The experiment was performed as previously described (21). In brief, an electric circuit model was used describing the epithelial properties: Epithelial resistance (R_epi_) consists of two parallel resistors, transcellular resistance (R_trans_) being further divided into resistors and capacitors, and the paracellular resistance reflecting the TJ formed resistance (R_para_). R_epi_ is in series to the subepithelial resistance (R_sub_), the latter caused by the filter support. IECs were grown on filter support and were mounted into a modified Ussing chamber setup and after application of alternating current (35 μA/cm^2^, frequency range 1.3 Hz to 65 kHz), voltage changes were detected by phase-sensitive amplifiers (402 frequency response analyzer, Beran Instruments, Glen Allen, VA, USA; 1,286 electrochemical interface; Solartron Schlumberger, Atlanta, GA, USA) and the resulting complex impedance values were calculated and plotted in a Nyquist diagram, which allowed to evaluate R_sub_ and R_epi_ (One-path impedance spectroscopy). R_trans_ and R_para_ (Two-path impedance spectroscopy) were determined from experiments in which the impedance spectra and fluxes of a paracellular marker substance, fluorescein, were obtained before and after chelating extracellular Ca^2+^ with EGTA. This caused TJs to open and to increase paracellular flux inversely proportional to R_epi_ changes.

### Sandwich Assay

The sandwich assay was done as previously described (22) and was performed at RT with cells growing on transwells (0.6 cm^2^, 0.4 μm pore size). IECs were washed in FBS-free medium and were incubated basolaterally with avidin (15 μM, 10 min). After washing with PBS^+^, cells were exposed to 140 μl of biotinylated dextran-3000-TexasRed (10 μM, 10 min, MolProbes) from the apical side. Cells were then fixed (2% PFA, 30 min, RT) and mounted for confocal microscopy.

### Calcium Switch Experiment

Experiments were done as previously described (23). Seven days after seeding them on PCF filters (0.4 μm), T84 cells were switched to a low calcium medium (DMEM calcium-free, Gibco) supplemented with 5% of FBS and 1% penicillin and streptomycin (Corning). To disrupt cell adhesion, cells were kept 16 h in low calcium medium after 4 times PBS washing in the presence or absence of IL-22 (10 ng/ml). Then, filters were mounted to Ussing chambers, where TER was monitored in 10 s-intervals throughout the experiment. After 30 min of equilibration, calcium chloride was added to both chamber sides at a final concentration of 1.6 mM for 6 h.

### Immunostaining and Confocal Laser Scanning Microscopy

Epithelial cell layers were washed 3× with PBS, then fixed with PFA 4% pH 7,5 and kept in 4°C with PBS for maximally 7 days prior to immunostaining. Cells were washed and stained following the protocol published previously (13) using the following primary antibodies: ZO-1 (1:100; BD Biosciences), JAM-A (1:100; Thermo Fisher). The secondary antibodies used were Alexa Fluor 488 goat anti-mouse or rabbit IgG, and Alexa Fluor 594 goat anti-mouse or rabbit IgG (1:500; Thermo Fisher). To determine occludin expression and cellular distribution, an occludin mouse monoclonal antibody (OC-3F10) was used as an Alexa Fluor® 594 Conjugate (Thermo Fisher). Nuclei were stained using DAPI (4′,6-Diamidin-2-phenylindol, conc. 1:2000). Immunofluorescence staining was analyzed by confocal laser scanning microscopy (LSM 780, Carl Zeiss, Jena).

### Migration and Invasion Assay

HT29/B6 cells were kept on at 37°C in a 5% CO_2_ environment until reach confluence. Subsequently, a defined scratch (diameter 100 μm) was introduced to filter-grown HT29/B6 cells and kept with medium with 1% of fetal bovine serum (Gibco) to avoid cell proliferation. Cells were exposed to IL-22 (10 ng/ml) and migration was evaluated by measuring the distance at 24 and 48 h after scratching. To perform invasion assay, Matrigel® was diluted (1 mg/ml), placed 100 μl into upper chamber of 24-well transwell and incubated at 37°C for 4–5 h. Subsequently, 2 × 10^5^ CaCo-2 cells in 100 μl plus 100 μl of media (MEM Eagle Medium, Gibco + 1% penicillin and streptomycin, Corning) without FBS were placed into the transwell chamber with Matrigel® cells were treated with or without IL-22 (100 ng/ml). In transwell lower chamber was added 600 μl of culture media and then, incubated for 24 h. The transwell chamber was removed and cells presented in the lower chamber were washed 2× with PBS^+^, fixed (PFA 2%) at room temperature for 30 min and stained with DAPI (1:2000 for 30 min). Number of colonies were counted and analyzed by confocal laser scanning microscopy (LSM 780, Carl Zeiss, Jena).

### Culturing 3D-Cysts, Immunostaining

For seeding CaCo-2 cells in Matrigel®, all materials were kept at 4°C under the cell culture bench. 1 × 10^4^ cells CaCo-2 cells were embedded in 150 μl of fluidic Matrigel® (Corning, Wiesbaden, Germany) prior to homogeneously seeding them to Lab-tek slides (Thermo Fisher). To allow the Matrigel® to consolidate, Lab-teks were incubated at 37°C for 30 min. Subsequently, 500 μl of Eagle's MEM, supplemented with 10% FBS (both Gibco/Thermo Fisher) was added. 3D-cysts evolved within 3 to 5 days (37°C, 5% CO_2_). Lab-teks were incubated at 4°C (PBS^+^) until immunostaining was performed. For immunostaining, cells were washed with PBS^+^ and then incubated with prewarmed collagenase (Sigma, Darmstadt, Germany; 8–10 min, 37°C), washed again and fixed using PFA (4%, pH 7.5) for 30 min at RT. Extensive PBS^+^ washes, then permeabilization/blocking using PBL-solution (0.7% fish skin gelatin and 0.025% saponin, in PBS^+^; 2 h, RT), followed by PBS-washes and quenching using 75 mM NH_4_Cl and 20 mM glycine in PBS^+^ (10 min, RT). Now, one wash using PBL and incubation with first antibody (PAR3 1:100; Sigma-Aldrich/ DLG1 1:100; Santa Cruz Biotechnology) in PBL was performed in a wet chamber overnight at 4°C. On the next day, samples were extensively washed using PBS^+^ at RT. Then incubation with the secondary antibody (in PBL, wet chamber, overnight, 4°C; Alexa594-Fab-fragment donkey anti-rabbit IgG, 1:200; Alexa488-Fab-fragment donkey anti-mouse IgG, 1:200, additionally phalloidin-Atto647, 1:200). Alternatively, antibody stainings with fluorescently tagged first antibodies were performed using a less complex protocol with overnight incubation of PFA-fixed 3D-cysts at 4°C with E-cadherin antibody (1:100; Alexa Fluor647-conjugate, BD Biosciences, San Jose, CA, USA) and DY-594-phalloidin (1:100; Dyomics, Jena, Germany) to stain actin. Nuclei were stained using DAPI (4′,6-Diamidin-2-phenylindol, 1:2000) for 1.5 h at RT. Microscopy was performed using a confocal laser scanning microscopy (LSM 780, Carl Zeiss, Jena).

### Treatment With Inhibitors

To inhibit STAT3 phosphorylation, different inhibitors were used. Stattic and STAT3 Inhibitor IV (S31-201) are cell-permeable molecules that inhibit by selectively binding the STAT3-SH2 domain impairing STAT3 activation, dimerization and nuclear translocation (24–26). Furthermore, it was used a cell-permeable peptide analog, which is also a selective blocker of STAT3 activation (27). As an indirect inhibitor, WP1066 was used that blocks STAT3 phosphorylation by binding to JAK2, a kinase upstream of STAT3 (28, 29). To inhibit the MAPK signaling, the inhibitor U0196 was used. It acts as a selective inhibitor of MEK1 and MEK2 preventing activation of MAP kinases p42 and p44 (ERK1/2) (30). Specifically, after 7 days in culture, HT29/B6 cells growing on transwell filters were exposed to the aforementioned STAT3 and MAPK inhibitors (Supplementary Table 1) for 2 h. Subsequently, IL-22 (10 ng/ml) was added for either 1 h after which cells were lysed, or for a maximum of 72 h for measuring TER (48 72 h) and cells were lysate to perform Western blotting experiments.

### RT-qPCR

Total RNA was extracted using the *mir*Vana™ mRNA Isolation Kit (Thermo Fisher) according to the manufacturer's instructions. To quantify the extracted RNA, NanoDrop 1000 (Thermo Fisher Waltham, MA, USA) was used. 800 to 1,000 ng of total RNA was applied to synthetize cDNA using the High Capacity cDNA Reverse Transcription Kit (Applied Biosystems/Thermo Fisher) according to the manufacturer's instructions. Real time-qPCR reactions were performed using 1 μl of cDNA template, 1 μl of the desired probe, 10 μL of RT-qPCR Master Mix (Applied Biosystems/Thermo Fisher) and nuclease-free water to a final volume of 20 μl. Comparative CT reactions were performed in triplicates using the StepOnePlus™ instrument (Applied Biosystems/Thermo Fisher). Calculations for gene expression changes were performed using the 2^−ΔΔCT^ method. The human probes used were all from Applied Biosystems/Thermo Fisher and were *SNAI1* (Hs00195591_m1), *SNAI2* (Hs00161904_m1), *MMP* −*2* (Hs01548727_m1), −*7* (Hs01042796_m1), and −*9* (Hs00957562_m1). *ACTB* (Hs01060665_g1) was used as control of the reaction amplification.

### Western Blotting

For protein quantification, epithelial cells were washed twice with ice-cold PBS^+^. Protein extraction was done using ice-cold lysis buffer (150 mM NaCl, 10 mM Tris buffer pH of 7.5, 0.5% Triton X-100, and 1% SDS). A volume of 10 ml lysis buffer was supplemented with one Complete Protease Inhibitor Cocktail tablet; Roche AG, Basel, Switzerland). Cells were scraped from the filters, incubated for 60 min on ice, and vortexed every 10 min. The supernatant was collected after centrifugation (30 min, 15,000 g, 4°C). To determine the protein content, Pierce BCA assay (Thermo Fisher, Waltham, MA, USA) was performed according to the product instructions using a Tecan plate reader (Tecan GmbH, Maennedorf, Switzerland) at an absorbance of 562 nm. Protein samples (20 μg) were mixed with 5× Laemmli buffer and loaded on premade SDS polyacrylamide gels (Bio-Rad, Feldkirchen, Germany). After electrophoretic separation, proteins were transferred to a PVDF membrane (Thermo Fisher) using the Trans-Blot system (Bio-Rad) at 25 V for 7 to 10 min and membranes were blocked for 2 h at RT with 1% PVP-40 (Polyvinylpyrrolidone; Sigma, Darmstadt, Germany) in TBST/0.05% Tween-20 buffer. Primary antibodies (Supplementary Table 2) were incubated overnight at 4°C. A peroxidase-conjugated secondary antibody was incubated (2 h, RT). Detection of proteins on the membrane was performed using SuperSignal West Pico Plus Stable Peroxide Solution (Thermo Fisher). Luminescent signals were detected with the Fusion FX7 imaging system (Vilber Lourmat Deutschland GmbH, Eberhardzell, Germany).

### Mice

Female WT and IL-22^−/−^ (on a C57BL/6 background), and NMRI mice were 8 to 12 weeks of age and bred and maintained in the Forschungsinstitut für Experimentelle Medizin (Charité—University Medicine, Berlin). Clinical conditions and body weights were determined daily, and all experiments were conducted according to the German animal protection laws. Animal protocols were approved by the Landesamt für Gesundheit und Soziales (Lageso, Berlin; TVV-No G0258/04).

### *Toxoplasma gondii*-Induced Ileitis *In vivo* Murine Model

Cysts of the *T. gondii* ME49 strain were obtained from brains of NMRI mice infected with 10 cysts for 2–3 months. Mice were infected with 100 cysts in 0.3 ml of PBS by gavage. All animal experiments were conducted according to the German animal protection laws. Histological scores and parasite loads were determined in formalin-fixed and paraffin-embedded tissue sections taken from the terminal ileum as described previously (31).

### Statistical Analysis

Statistical analysis was performed using GraphPad Prism software (GraphPad Software, La Jolla, CA) by the non-parametric Mann Whitney U test. All data are expressed as mean values ± standard error of the mean (SEM). *p* < 0.05 was considered significant.

## Results

### IL-22 Impairs Paracellular Intestinal Epithelial Barrier Integrity

**Figure 1:**
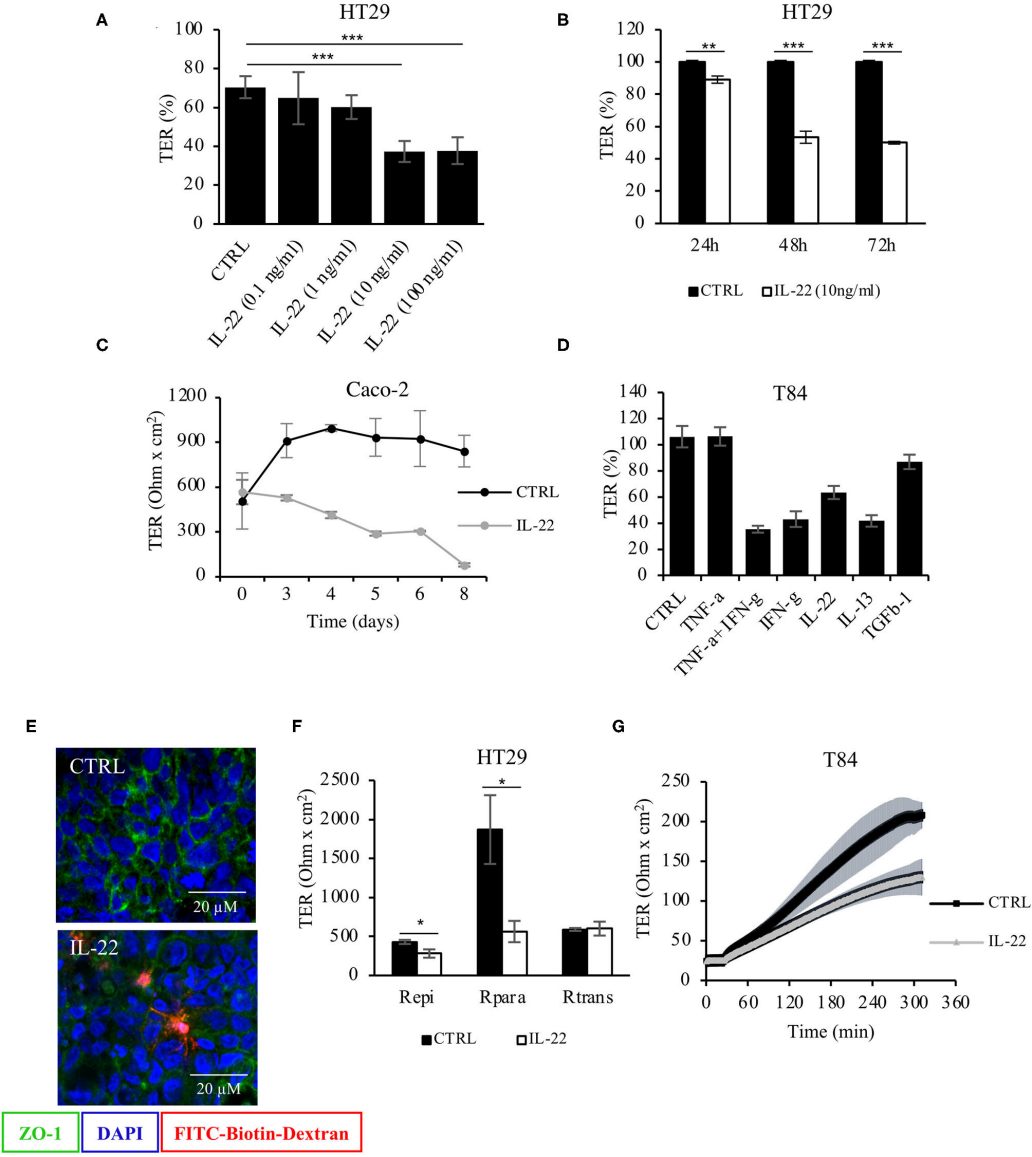
Barrier integrity is affected by IL-22. **(A)** Transepithelial resistance (TER) was determined in HT-29/B6 cells grown on transwell filters. Cells were exposed to IL-22 at different concentrations (0.1, 1, 10, and 100 ng/ml). TERs after 72 h of IL-22 exposure are shown *n* = 25. Mann–Whitney *U* test; ***p < 0.001. **(B)** TER time course in HT29/B6 cells exposed to IL-22 (10 ng/ml); *n* = 32. Mann–Whitney *U* test; **p < 0.01; ***p < 0.001. **(C)** TER measured in CaCo-2 cells exposed to IL-22 (10 ng/ml) for a longer time course (up to 8 days); *n* = 3. **(D)** Comparative analysis of TER in T84 cells (grown on transwell filters) after a 48 h-exposure to various cytokines (TNFα: 1,000 U/ml, IFNγ: 100 U/ml, IL-22: 10 ng/ml; IL-13: 10 ng/ml; TGF-b1: 20 ng/ml); *n* = 8. **(E)** Sandwich assay revealing transepithelial passage of macromolecules, specifically TexasRed-dextran3000 (red fluorescence) in control and IL-22-treated CaCo-2 cells. E-cadherin, green; nuclei, blue; *n* = 3. **(F)** Two-path impedance analysis: HT-29/B6 cells grown on transwell filters were exposed to IL-22 (10 ng/ml) for 48 h. After mounting filters to Ussing chambers paracellular and transcellular components of TER were determined by two-path impedance; *n* = 6. Mann Whitney *U* test; *p < 0.05 **(G)** Calcium switch experiment: T84 cells growing on transwell filters were exposed to IL-22 (10 ng/ml, 48 h) and mounted to Ussing chambers, where TER was monitored in 10 s-intervals throughout the experiment. Transepithelial resistance was measured every 60 min for 6 h; *n* = 3. Mann–Whitney *U*-test; *p < 0.05; **p < 0.01; ***p < 0.001.

To investigate the role of IL-22 on barrier integrity, intestinal epithelial cells (IECs) seeded on transwell filters were exposed to IL-22 (apical and basolateral compartment). A stable epithelial barrier was established in CaCo-2 cells on day 10, in HT-29/B6 cells on day 7 and in T84 cells on day 14. Subsequently, apical and basolateral cell surfaces were exposed to IL-22. Transepithelial electrical resistance (TER) was monitored throughout the experiment (Figure 1). IL-22 induced a significant decrease in TER in a dose-dependent (Figure 1A) and time dependent manner (Figures 1B,C) with reductions in TER as much as 60% of control level at 10 and 100 ng/ml of IL-22 (72 h exposure). Furthermore, IL-22-induced TER decrease was similar to that after 48 hours of exposure to other proinflammatory cytokines (Figure 1D). Interestingly, the IL-22-induced barrier leak also allowed the passage of macromolecules like TMR-dextran3000 as shown by the sandwich assay (Figure 1E). Furthermore, a 2-path-impedance analysis showed that the barrier leak occurred exclusively paracellular (Figure 1F). In addition, we observed an IL-22-associated delay of TER-recovery after switching the media from calcium-free to normal calcium concentrations in CaCo-2 cell layers in filter transwells that had been mounted to Ussing chambers. This finding is frequently found with a disturbed TJ assembly (Figure 1G). Altogether, these results show that IL-22 impairs the paracellular barrier function of IECs and promotes an increased permeability of small ions (measured by TER) and macromolecules (as measured by the sandwich assay).

### IL-22 Induces Defective Epithelial Polarity

**Figure 2:**
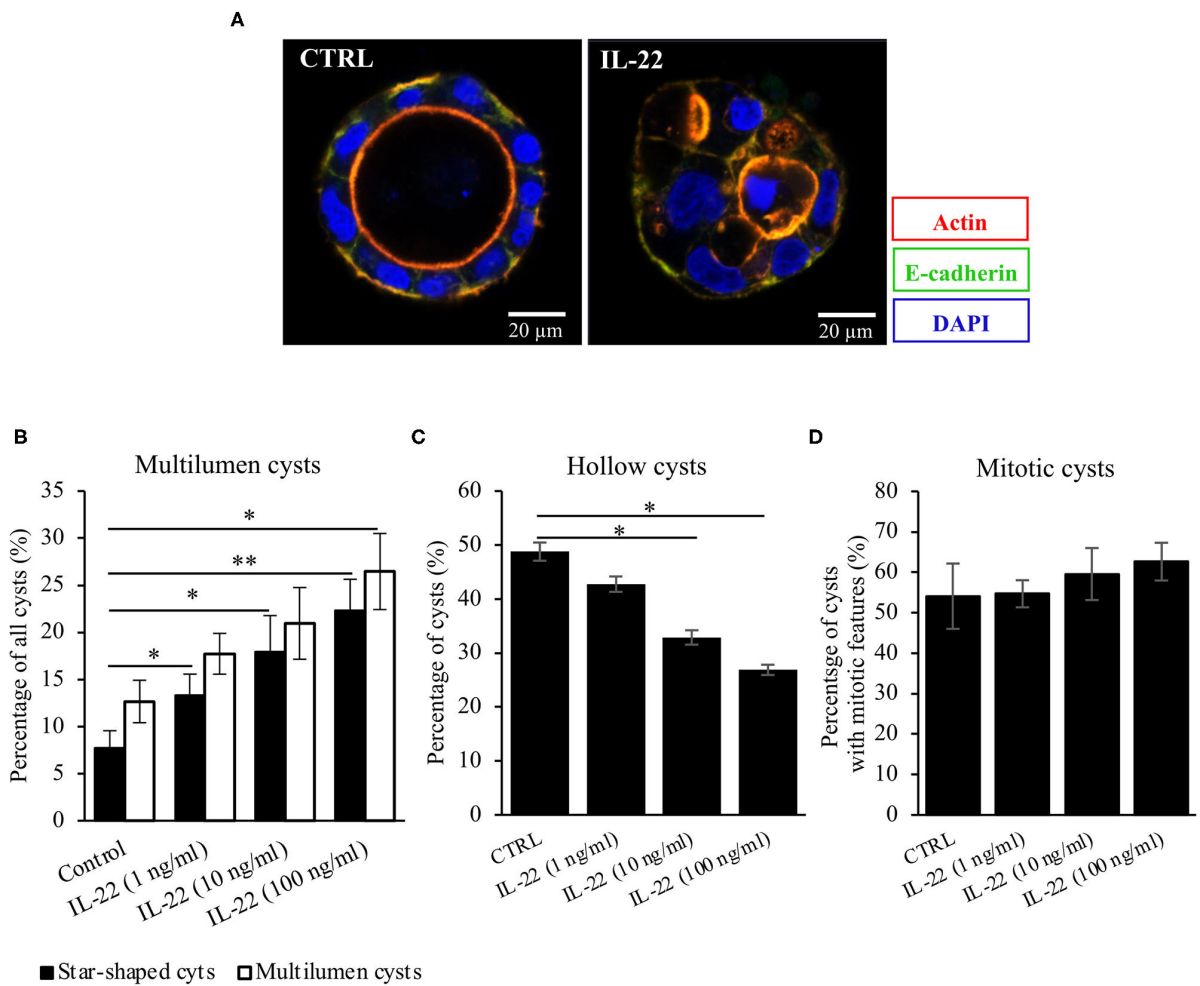
IL-22 exposure causes atypical cysts formation. **(A)** CaCo-2 cells were seeded in Matrigel® and grown for 5–7 days to form 3D cysts. Subsequently, they were fixed and immunostained. Blue, nuclei; red, actin; green, E-cadherin. Representative images *n* = 6. **(B–D)** Quantification of the 3D-cyst experiments: CaCo-2 cysts growing in Matrigel® were analyzed by confocal LSM. Multilumen, hollow cysts and cysts revealing mitoses were microscopically quantified; *n* = 6. Mann–Whitney *U*-test; *p < 0.05; **p < 0.01.

**Figure 3:**
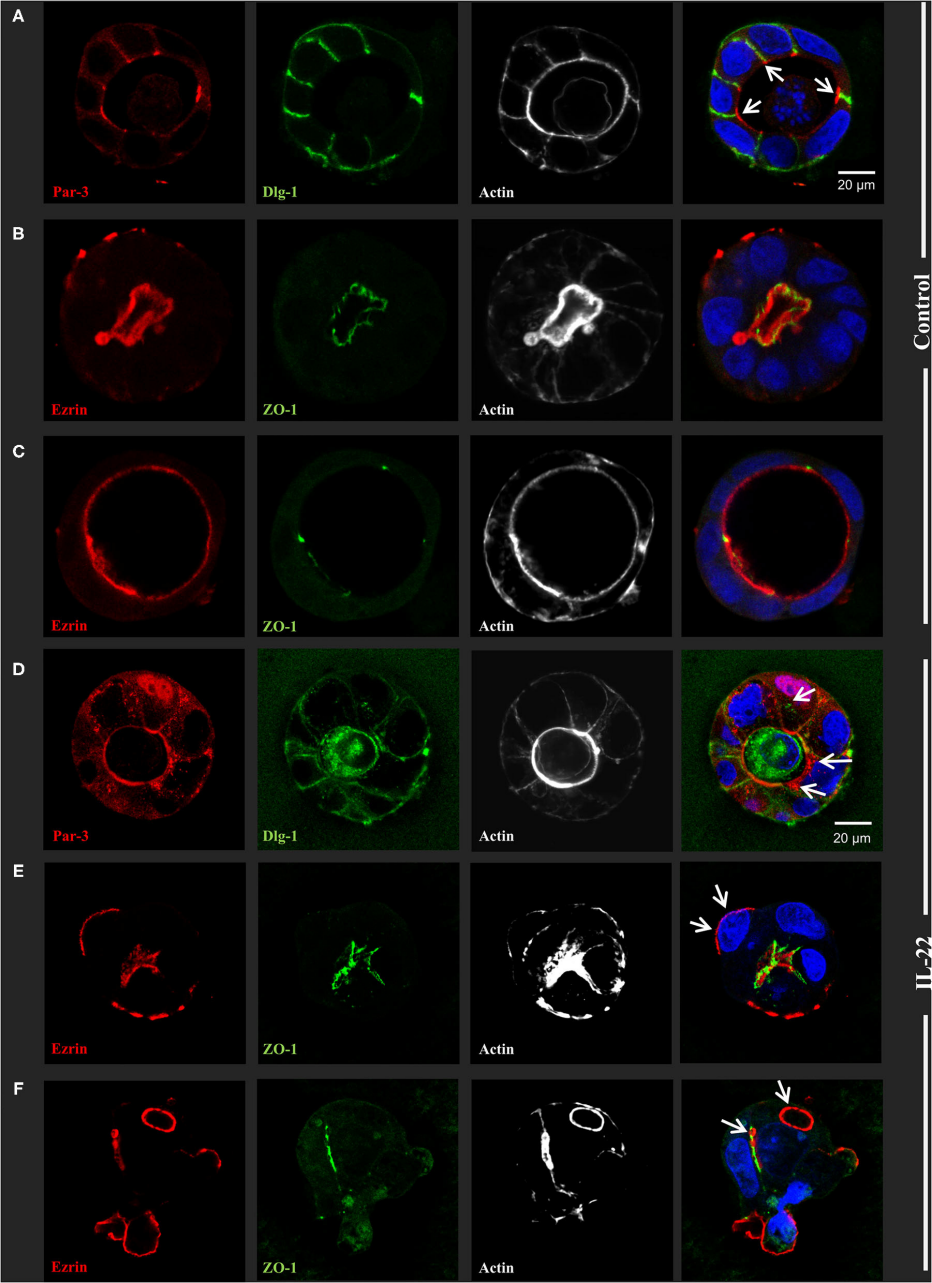
IL-22 induces development of dyspolar 3dimensional cysts. CaCo-2 cells were seeded in Matrigel and 3D-cysts were allowed to develop within 5–7 days. Then cysts were fixed and immunostained according to the Methods section. **(A–C)** control 3D-cysts. **(D–F)** cysts treated with IL-22 (10 ng/ml) starting at the day after seeding. Proteins detected by immunostaining are depicted in each image. The composite image (right column) additionally includes staining for nuclei using DAPI. Structures identified by arrows are explained in the text of the Results chapter; *n* = 4 independent experiments.

Next, CaCo-2 cells seeded in Matrigel® were allowed to evolve to 3-dimensional cysts. Formation of cyst lumen was analyzed as this is known to reflect the integrity of the polarization process. Cells were then immunostained and analyzed by confocal laser scanning microscopy. Untreated CaCo-2 3D cysts most often exhibited a single lumen, lined with a single epithelial layer with the apical cell surface pointing to the lumen and the basolateral surface pointing to the Matrigel®-containing matrix. In untreated cysts, phalloidin staining showed a strongly stained subapical network of actin fibers, while the basolateral membrane was E-cadherin as well as β-catenin-positive as expected in polarized IECs (Figure 2A, Supplementary Figure 1). Interestingly, exposing cysts to IL-22 resulted in an increase of cysts with multiple lumens and a consecutive decrease of hollow cysts, i.e. cysts displaying a single, “ball-shaped” lumen (Figures 2B,C). In this regard, IL-22-treated cysts frequently revealed dystopic lumen formation, e.g., in between neighboring IECs of the single cell lining of the cysts (Figure 2A, Supplementary Figures 1E, 2C). Nevertheless, the number of cysts with mitotic spindles was not significantly changed upon IL-22 treatment (Figure 2D). Furthermore, we immunostained key cell polarity proteins, including Par-3, that has been described to orchestrate the assembly of apical junctions in epithelial cells and was thus expected to localize to TJs in polarized IECs, ZO-1 as a protein localizing to TJs, Ezrin as a component of the apical membrane and Dlg-1, demarcating the basolateral membrane. In general, we confirmed the expected protein localizations in established cysts 5 days after seeding (Figures 3A–C). Par-3 was localized to the most apical part of the lateral cell membrane in control cysts revealing the same localization as ZO-1 (Figure 3A, arrows; Supplementary Figures 2A,B). Ezrin was associated with the apical membrane and Dlg1 was restricted to the basolateral membrane. In contrast to that, in IL-22-treated cysts Par-3 was dislocated as it was found diffusely along the entire lateral membrane and also in intracellular vesicles (Figure 3D). Furthermore, membranous Dlg-1 staining was reduced compared to controls and was shifted to an intracellular compartment (Figure 3D). Ezrin staining was focally enriched at the basal membrane (instead of the apical membrane, Figure 3E, arrows), suggesting opposite polarization. In other cysts it demarcated aberrant lumens (Figure 3F, arrows). Taken together, these results suggest that IL-22 impairs intestinal epithelial polarity and lumen formation.

### IL-22 Increases Cell Motility, Cell Invasion, and Induces EMT

**Figure 4:**
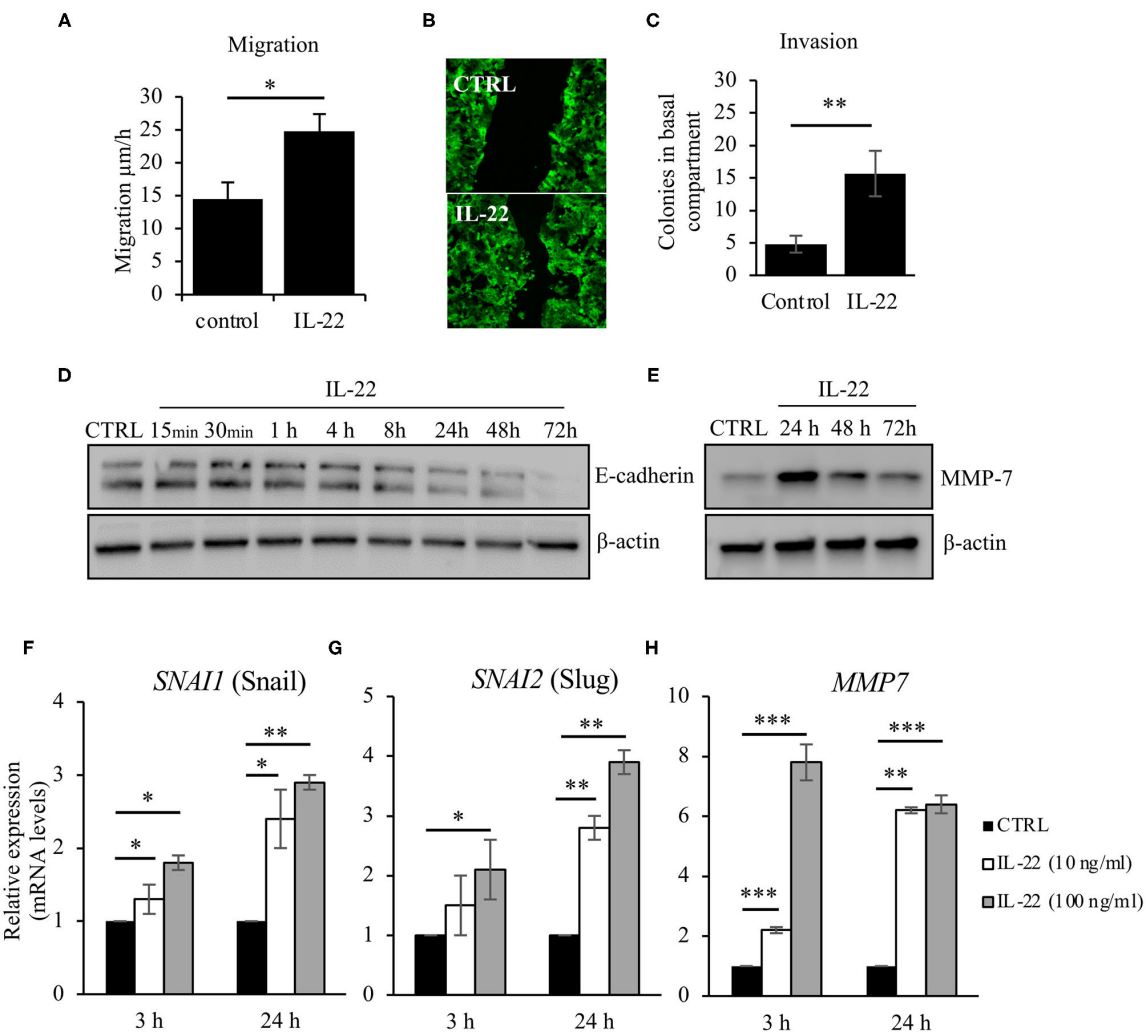
IL-22 increases cell motility and induces EMT on IECs. **(A–C)** HT29/B6 cells exposed to IL-22 (10 ng/ml) growing on transwell filters were scratched (diameter 100 μm). Migration was evaluated by measuring the remaining scratch width at 24 and 48 h after scratching by fluorescence microscopy; *n* = 3 **(B)** For invasion, CaCo-2 cells were seeded on Matrigel®-coated filters and then exposed to IL-22 (100 ng/ml). The invasion of CaCo-2 cells through the Matrigel®-coated filter was quantified by counting colonies that formed on the basal side of the filter *n* = 3. **(D,E)** CaCo-2 cells were exposed to IL-22 (10 ng/ml) and lysed. E-cadherin and MMP-7 protein levels were investigated through western blotting. Representative Western blots of three and two independent experiments, respectively. **(F–H)** CaCo-2 cells were exposed to IL-22 (10 or 100 ng/ml) for 3 and 24 h. RNA was extracted and RT-qPCR was performed to quantify expression of Snail (SNAI1), Slug (SNAI2), and MMP-7. Expression levels were calculated using the 2^−ΔΔCT^ method. Mann–Whitney *U*-test; *n* = 3; *p < 0.05; **p < 0.01; ***p < 0.001.

As we had observed IEC polarity defects after IL-22 exposure, we next asked, whether IL-22 might also impact migratory and invasive properties of IECs. Thus, we carried out a CaCo-2 wound healing assay by performing uniform scratches into a single CaCo-2 layer that stably expressed Actin-GFP and monitored live by confocal LSM. Exposure to IL-22 (10 ng/ml) resulted in a statistically significant increased IEC migration, thereby nearly doubling IEC migratory speed (Figures 4A,B). Similarly, IL-22 had the capacity to induce invasion of cells in a combined Matrigel®/filter-based assay. After IL-22 exposure, the number of invaded colonies was ~3-fold higher compared to control cells (Figure 4C). To us, these results appeared to be plausible findings in the context of epithelial-to-mesenchymal transition (EMT). Thus, the following experiments were designed to assess whether IL-22 induces EMT in IECs. Firstly, levels for proteins that are regulated within the EMT process, specifically E-cadherin and matrix metalloprotease-7 (MMP7), were quantified by western blotting in the course of exposing IECs to IL-22. While E-cadherin levels declined starting between 4 and 8 h of IL-22 exposure continuously, MMP7 expression peaked 24 h after IL-22 addition (Figures 4D,E). To further support the hypothesis that an EMT program is induced by IL-22, mRNA levels of classical EMT transcription factors, *SNAI1* (Snail) and *SNAI2* (Slug), were assessed after exposing the cells for 3 and 24 h to IL-22 (10 and 100 ng/ml). IL-22 significantly increased *SNAI1* and *SNAI2* gene expression at 24 h even higher than at 3 h of IL-22 exposure at both IL-22 concentrations (Figures 4F,G). In addition, MMP7-RNA levels were strongly upregulated after 3 and 24 h of IL-22 exposure (Figure 4H), in accordance to our previous data showed on protein levels by western blotting. In summary, these data indicate that IL-22 induces an EMT-like cell program, which might contribute to migratory as well as invasive properties of IL-22-treated IECs.

### IL-22 Modulates Tight Junction Protein Expression

**Figure 5:**
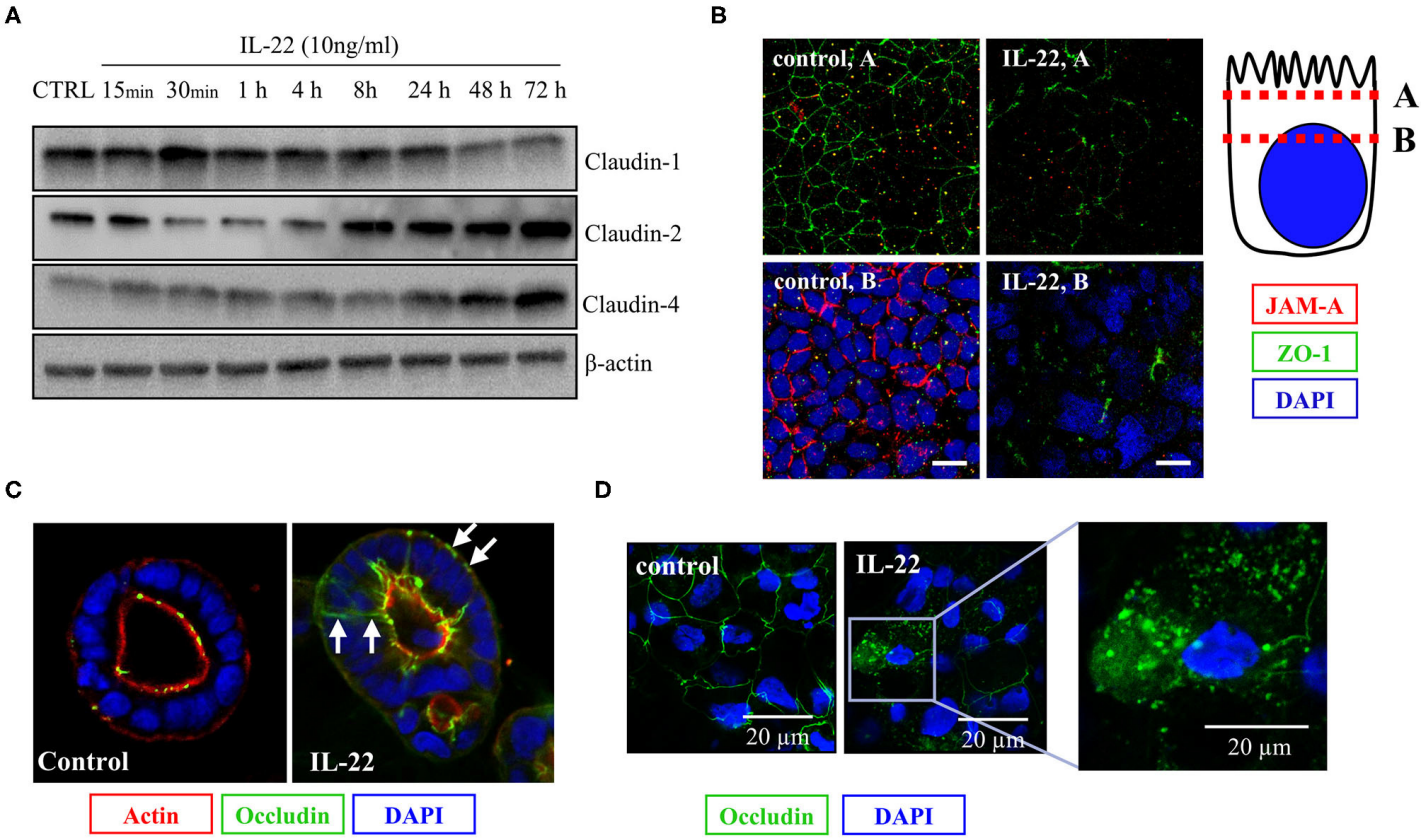
IL-22 affects tight junction proteins. **(A)** Expression of TJ proteins: IECs were treated with IL-22 (10 ng/ml) for the indicated times. Subsequently, cells were lysed. TJ protein levels were determined by Western blotting as explained in the Methods section. Representative blots of three independent experiments. **(B–D)** Confocal LSM after immunostaining of CaCo-2 cells. **(B)** Reduction of JAM-A junctional levels by IL-22. Red, JAM-A; green, ZO-1; blue, nuclei *n* = 3. IECs cartoon represents the confocal microscopy analysis **(C,D)** Dislocation of occludin by IL-22. Green, occludin; red, actin; blue, nuclei; *n* = 3.

As we had shown that IL-22 induces a paracellular barrier defect in IECs and modulates the expression of genes that regulate junctional proteins, we next investigated the impact of IL-22 on expression and subcellular localization of TJ proteins. In a first step, we monitored the expression of various TJal claudins in the course of IL-22-exposure by western blotting (Figure 5A). As early as 4 h after exposing the cells to IL-22, claudin-1, a barrier-forming claudin, decreased on the protein level, whereas protein levels of the pore-forming claudin-2 and claudin-4, which was previously linked to EMT were increased (Figure 5A). Using confocal LSM we moreover found, that the PDZ-containing TJ-associated protein ZO-1 as well as the junctional adhesion molecule-A (JAM-A) were reduced in their junctional expression (Figure 5B). Similarly, TJal localization of occludin was shifted to an intracellular and a lateral membrane localization in the 2D transwell and the 3D cyst model, respectively (Figures 5C,D).

### IL-22 Mediates TJal Barrier Defects via ERK Pathway

**Figure 6:**
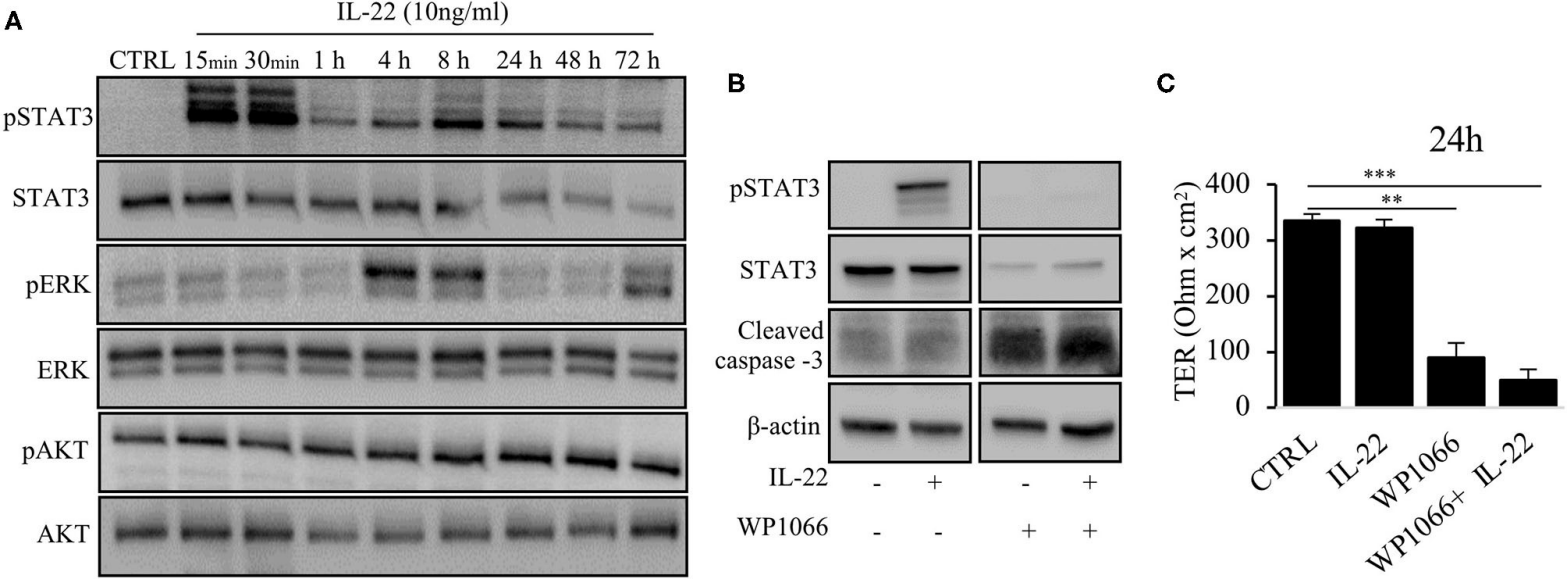
IL-22 induces STAT3 and ERK phosphorylation. **(A)** HT-29/B6 cells were exposed to IL-22 (10 ng/ml) for the indicated times. Subsequently, cells were lysed and protein levels of STAT3, ERK and AKT and their phospho-levels were investigated by Western blotting. Representative blots of three independent experiments are shown. **(B)** HT-29/B6 cells were incubated in the presence of the STAT3 inhibitor WP1066 (50 μM) for 2 h and IL-22 (10 ng/ml) for 1 h as indicated. Western blotting of cell lysates was performed to quantify protein levels of STAT3 total, phospho-STAT3 (pSTAT3), cleaved caspase-3, and b-actin as loading control. **(C)** TER was determined after 24 h of IL-22 exposure of HT-29/B6 cells growing on transwell filters treated with IL-22 (10 ng/ml), WP1066 (50 μM) as indicated. Mann–Whitney *U* test; *n* = 3; *p < 0.05; **p < 0.01; ***p < 0.001.

**Figure 7:**
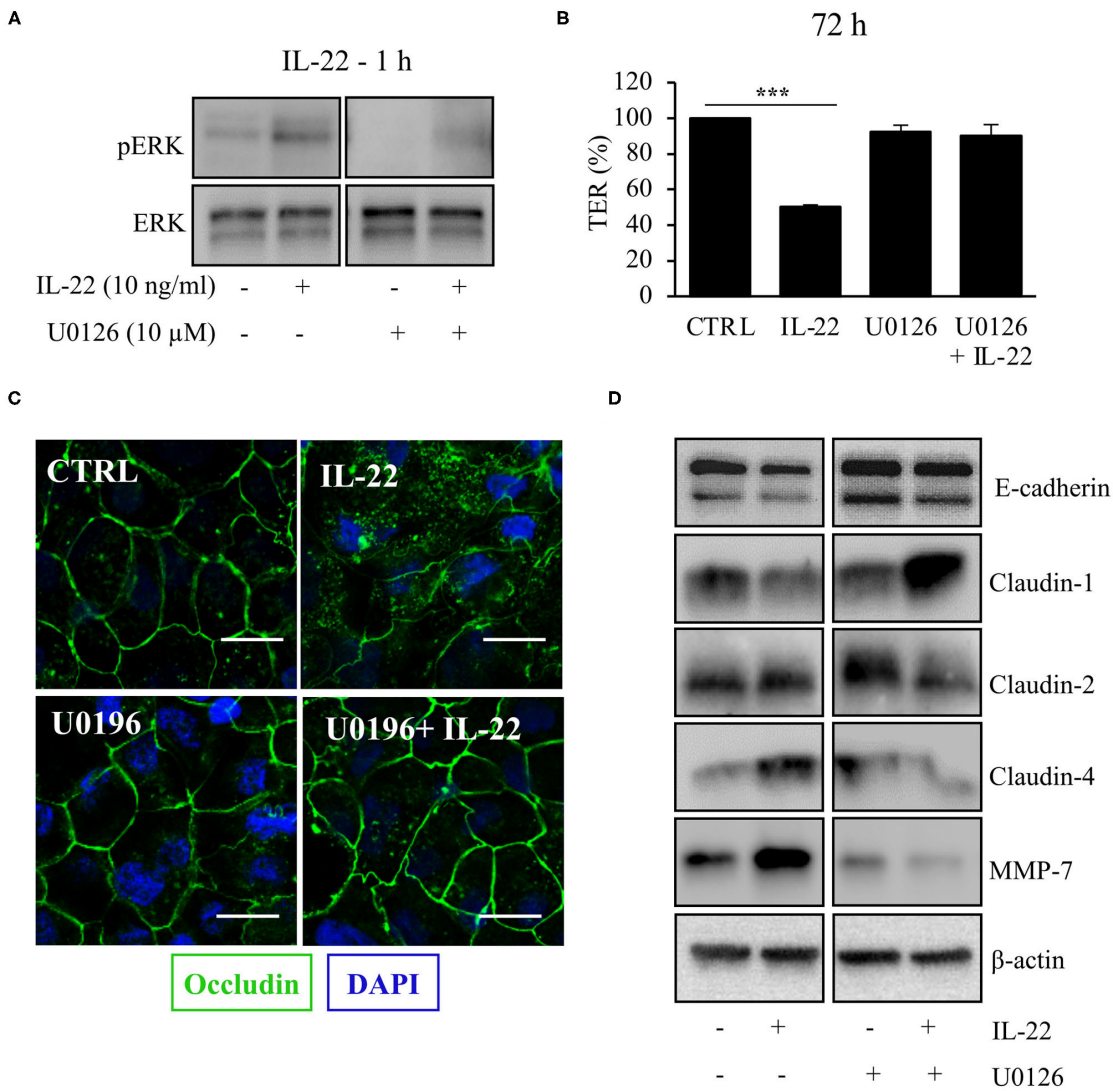
MAPK/ERK signaling pathway is pivotal for IL-22 barrier signaling in IECs. HT-29/B6 cells were exposed to U0196 (2 h) in the presence or absence of IL-22 (1 h). **(A)** Protein levels of ERK and phospho-ERK were assessed by Western blotting. Representative blots of three independent experiments are shown. **(B)** TER of HT-29/B6 cells were measured after treatment with U0126 and IL-22 (10 ng/ml), as depicted; *n* = 9, ***p < 0.001. **(C)** Confocal LSM after immunostaining of occludin was performed. Green, occludin; blue, nuclei. Scale bar: 20 μm. Representative images of three independent experiments. **(D)** Western blotting was performed to quantify protein levels of E-cadherin, claudin-1,−2,−4, and MMP-7. Representative blots of two independent experiments are shown; ***p < 0.001.

Next, we aimed to dissect the intracellular signaling pathways after activation of the IL-22 receptor in IECs. All three IEC cell lines involved in this study equally expressed the two IL-22 receptor subunits (IL-22Ra1 and IL-10Rb), but did not express the endogenous IL-22 antagonist, the IL-22 binding protein, IL-22BP (Supplementary Figure 3). As shown in various previous studies, upon IL-22 receptor activation the STAT3 pathway as well as the MAPK/ERK pathway were activated. However, this occurred non-simultaneously (STAT3 at 15 min, ERK between 30 min and 4 h, Figure 6A). Interestingly, we did not detect any phosphorylation of AKT in our model system. Since activation of STAT3 signaling was previously reported to play a role in epithelial protection, we next determined the effect of various strategies to inhibit STAT3 signaling on STAT3 activation and epithelial barrier function (Figures 6B,C). While the STAT3 inhibitor WP1066 reduced the pSTAT3 signal, it also reduced total STAT3 levels which was explained by a strong induction of programmed cell death (as revealed by cleaved caspase-3, Figure 6B). Consecutively, STAT3 inhibition did not rescue the IL-22-induced barrier defect (Figure 6C). In line with this finding other STAT3 inhibitors were not capable of reversing epithelial barrier defects by IL-22 (Supplementary Figure 4). On the other hand, inhibition of ERK/MAPK was successful regarding signaling as well as rescue of barrier function (Figures 7A–D). Using the MEK inhibitor U0126, we achieved close to total inhibition of ERK phosphorylation, thereby rescuing the IL-22-induced TER-reduction (Figures 7A,B). In accordance with the signaling study, IL-22-induced dislocation of occludin was reversed by MAPK inhibition (Figure 7C). Similarly, reduction of E-cadherin and claudin-1 protein levels as well as increases in claudin-2,−4 and MMP7 were normalized by U0126 treatment (Figure 7D). Altogether, our results indicate that MAPK/ERK signaling is central in mediating IL-22-dependent barrier and EMT signaling in IECs.

### IL-22 Induces Barrier Defect in a Mouse Model of Terminal Ileitis

**Figure 8:**
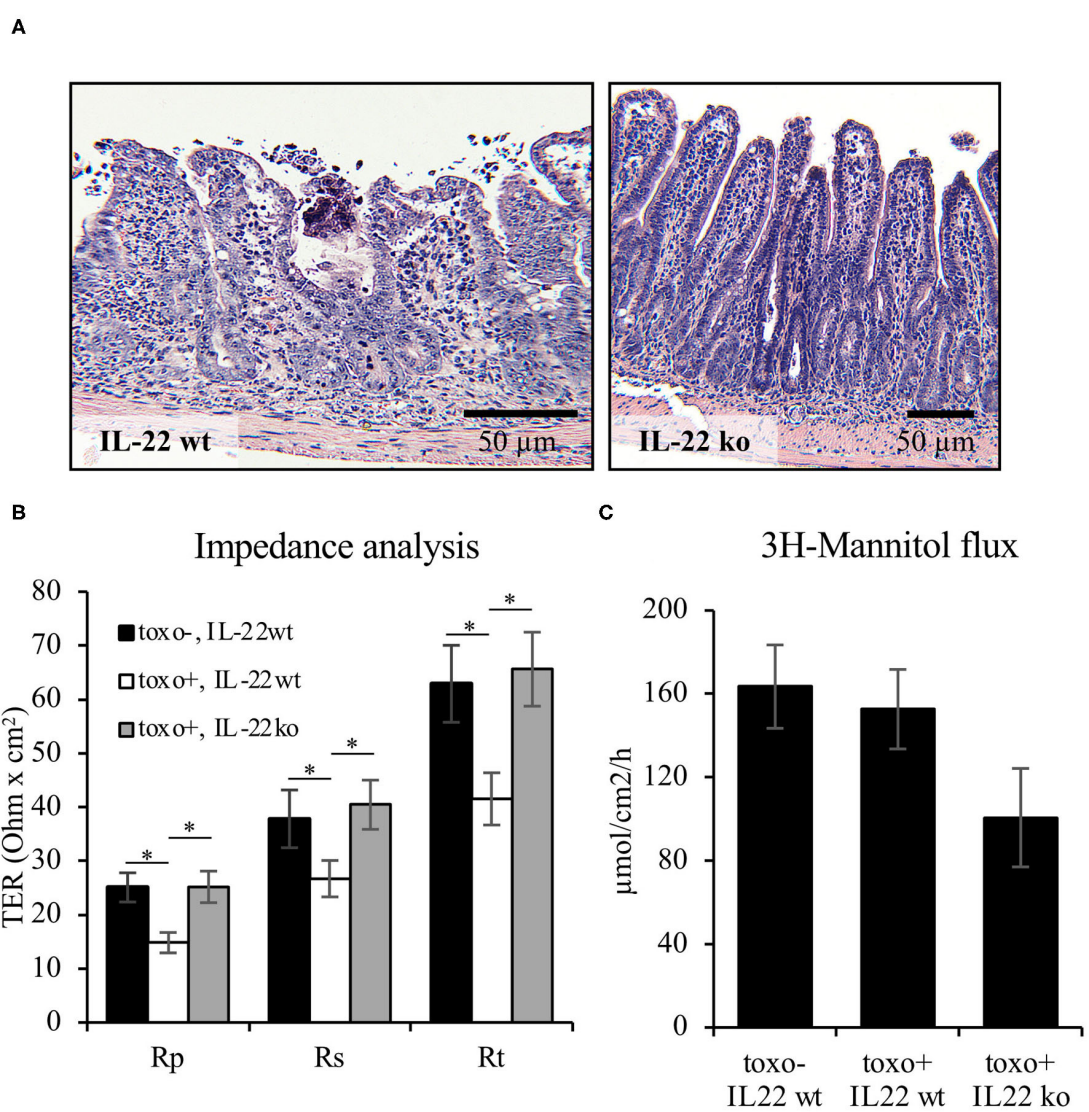
IL-22 induces an epithelial barrier defect in murine mucosa. For induction of a murine *T. gondii* terminal ileitis, IL-22-deficient and wildtype control mice were orally infected with T. gondii cysts. The terminal ileum was explanted at day 7. **(A)** Histological examinations were done in formalin-fixed and paraffin-embedded tissue following H&E staining. **(B,C)** Explanted mucosae from the murine terminal ileum were mounted to Ussing chambers and analyzed by one-path impedance spectroscopy to examine total intestinal wall resistance (R^t^), the epithelial resistance (R^e^) and the subepithelial resistance [R^s^, **(B)**]. Macromolecular permeability was determined by measuring the flux of 3H-mannitol **(C)**; *n* = 9, *p < 0.05.

In addition to the IEC *in vitro* experiments, barrier function was examined using the *Toxoplasma gondii* (*T. gondii*) mouse model of terminal ileitis in mice lacking IL-22 (8). *T. gondii* has previously been described to induce IL18 expression in IECs in an IL22-dependent manner. However, the detailed consequences on the structural and functional intestinal barrier mediated by the presence or absence of IL22 have not been investigated. In line with previously published data, *T. gondii* induced a severe terminal ileitis in C57Bl/6 mice after seven days of infection as seen in H&E stainings of formalin-fixed, paraffin-embedded sections (Figure 8A). Mucosae from the terminal ileum of IL-22 deficient and wild type control mice were mounted to Ussing chambers and analyzed by one-path impedance spectroscopy to examine not only the total intestinal wall resistance, but also its epithelial portion [R^e^, (15), Figure 8B]. As expected, the terminal ileal mucosa of *T. gondii*-infected wild type mice displayed a significant defect of the epithelial barrier and an increase in macromolecular permeability when compared to wildtype mice (3H-mannitol, Figures 8B,C). Interestingly, in line with our cell culture findings, mice lacking IL-22 expression were protected from this barrier defect and exhibited a significantly higher R^e^ and a statistically non-significant tendency toward a higher mannitol permeability (Figures 8B,C). In summary, these results give *in vivo* and *ex vivo* evidence showing that IL-22 plays an important role in the intestinal barrier function.

## Discussion

IL-22 has a central role in type 3 mucosal immunity, which is directed against extracellular bacteria and fungi. IL-22 is tonically secreted by ILC3 cells sedentary to the gut mucosa and, additionally, “on demand” by T_H_17 cells (10, 32). The fact, that the IL-22 receptor is exclusively expressed on non-hematopoietic epithelial and stromal cells, has prompted the idea of an ILC3-IL-22-IEC axis (32). Current understanding implies that IL-22 is contributing to type 3 immunity by (*ex vivo*) the production of antimicrobial peptides (AMPs) including β-defensins, the C-type lectins RegIIIβ and RegIIIγ, lipocalin-2, and calprotectin as well as amplifying IEC turnover to disturb colonization of the gut epithelial lining by bacteria (10, 12, 33–35). Furthermore, it has been described that IL-22 supports “epithelial integrity,” which however, is incompletely analyzed so far, since studies have mostly focused on AMP expression and wound healing assays and missed out classical barrier function as well as analysis of the apical junctional complex (7, 17). Thus, our study aimed to functionally analyze the IL-22-exposed epithelial barrier and to characterize IL-22-altered epithelial polarity as this might be fundamental to orchestrating barrier function (36, 37).

Our first finding—IL-22 inducing a profound reduction of IEC transepithelial resistance corresponding to a relevant increase in small ion flux—came unexpected as it is common knowledge that IL-22 rather stabilizes but destroys epithelial barrier function (7, 17). Since it was unexpected, we validated this finding in three different intestinal epithelial cell lines, established its dose- and time-dependence and presented evidence that these findings correspond to the IL-22-related epithelial barrier function in murine intestines as *ex vivo* one-path impedance analyses of mucosae from IL-22 knock-out mice revealed an IL-22-dependent epithelial barrier defect. Importantly this defect (i) is clearly localized to the paracellular junction as evaluated by two-path impedance analysis and (ii) is not limited to small ion flux as shown by sandwich assay studies and by a tendency to higher mannitol fluxes in the Ussing studies on murine intestinal epithelia. Nevertheless, two studies, specifically those by Tsai et al. (38) and by Wang et al. (39) are in full accordance to our findings, since both uncovered an IL-22- and claudin-2-dependent mechanism for triggering a leak-flux diarrhea in the murine *Citrobacter rodentium* and an epithelial barrier defect for small solutes in the CaCo-2BBE model.

After having confirmed that the previously described wound healing potential of IL-22 holds also true in our model, we questioned, whether IL-22 might reprogram epithelia in a way that would explain likewise the induction of transient increases in solute permeability and the potential to support the healing of mucosal wounds. Our working hypothesis was that this would be compatible with epithelial-to-mesenchymal transition (EMT). Hence, we established an IL-22-induced expression of transcription factors (Snail, Slug) characteristic for EMT, as well as decreased expression of epithelial markers (E-cadherin) as well as induction of a protein that points to a reorganization of mucosal architecture and allows for epithelial invasion (MMP7). IL-22's potential to induce EMT can be compared to that of IL13 as this T_H_2 cytokine had been previously shown to induce EMT in a similar fashion (40). However, if an EMT-like program orchestrates the reorganization of TJs aiming to release junctional tightness and thereby facilitating IEC migration into a wound, epithelial polarity is likely altered beforehand (41). Thus, we investigated the status of epithelial polarity after exposing intestinal epithelial cysts with IL-22. Indeed, IL-22 significantly disturbed epithelial polarity including the establishment of a single lumen in Caco-2-cysts as well as the dislocation of polarity complex proteins that (like Par3) are pivotal to the assembly of primordial apical junctions. Accordingly, TER monitoring of IL-22-exposed IECs after calcium switch provided functional evidence for a defective assembly of TJs.

In terms of intracellular signaling the study was in contradiction to a number of previous studies as it did not confirm the prominent role of JAK/STAT signaling, especially STAT3, in our model system (7, 17, 38, 39). In fact, STAT3 was activated by IL-22 exposure but could only be related to survival signaling and not to TJal and polarity reprogramming. Instead, we found a mostly unprecedented function for IL-22-induced activation of MAPK, since inhibitor studies revealed evidence for ERK signaling to be causative for signaling to TJs. Our experiences, that epithelial cell death occurs as soon as STAT3 is inhibited goes in line with results from several previous publications (17, 42). One should emphasize the limitation that our study was performed using cell lines and that a consecutive study using primary cells, e.g., a 2D organoid model, might help to solve this controversy.

Moreover, our data suggest that the potential of IL-22 to reprogram epithelia in order to close mucosal wounds comes with the expense of acquiring dyspolar epithelia and to induce cellular features as MMP7 expression and actin filaments-driven invadopodes that finally contribute to epithelial invasiveness. This goes in line with data on murine colitis-associated cancer, which, however, reveal some complexity (42). On the one hand, IL-22-knock-out mice develop a higher tumor burden, which was related to a substantially increased inflammatory activity after colitis induction by dextrane sulfate sodium. On the other hand, mice, in which the endogenous IL-22 opponent, IL-22-BP, was knocked out also developed more tumors, which was interpreted as a long-term effect of the increased IL-22-availability and secondary to that prolonged epithelial proliferation during the recovery phase of inflammation (43, 44).

In summary, we have used model systems including three IEC lines, functional as well as subcellular structural experimental setups and a murine terminal ileitis model to describe the epithelial response to the T_H_17 cytokine IL-22. From these data we propose that IL-22 induces an EMT-like program that induces intestinal epithelia to reduce their epithelial-specific polarity. Secondary to that, a loosening of intercellular junctions occurs, that allows IECs to migrate into wounds but also to become more invasive. It is so-far speculative, that the latter process might contribute to colitis-associated carcinogenesis once the IL-22:IL-22BP ratio becomes too high. Interestingly, we found rather MAPK/ERK to be responsible for these actions than the JAK/STAT pathway.

## Data Availability Statement

The original contributions presented in the study are included in the article/Supplementary Material, further inquiries can be directed to the corresponding author/s.

## Ethics Statement

The animal study was reviewed and approved by Landesamt für Gesundheit und Soziales (Lageso, Berlin; TVV-No G0258/04).

## Author Contributions

DD: plan and carry out the signaling studies and barrier experiments, confocal LSM, and writing the paper. LL: plan and carry out the 3D cyst studies and barrier studies, and confocal LSM. DC-S: carry out the EMT studies and western blotting. VD: western blotting of STAT3 inhibitor studies. SK: plan and carry out 2-path impedance analysis and revision of manuscript. JR: design of the 3D cyst assay and the sandwich assay. CH: immunostaining and cell culture. KW and RS: planning and carrying out of IL22 and IL22 receptor RT-PCR. SM: paper writing and statistics. MM and MH: establish and generate the terminal ileitis mouse model. BS: experimental strategy and writing the paper. MS: defining the experimental strategy, barrier studies on mouse mucosa, 3D cyst assays, immunostaining and confocal LSM, and writing the paper. All authors contributed to the article and approved the submitted version.

## Conflict of Interest

The authors declare that the research was conducted in the absence of any commercial or financial relationships that could be construed as a potential conflict of interest.
