# Supplementary material for: Reprogramming Intestinal Epithelial Cell Polarity by Interleukin-22
Source: Front Med (Lausanne). 2021 Apr 12;8:656047. doi: 10.3389/fmed.2021.656047 (PMC8072225; doi:10.3389/fmed.2021.656047)
Supplement: Supplementary file 1 [file Data_Sheet_1.PDF]

Supplementary Material

Overview

1 Supplementary Figures

1.1 Suppl. figure 1

1.2 Suppl. figure 2

1.3 Suppl. figure 3

1.4 Suppl. figure 4

2 Videos

2.1 Video-1, untreated Caco2- cyst revealing a single lumen. Red, actin; green, E-cadherin; blue, nuclei

2.2 Video-2, Multilumen cyst (IL-22, 10 ng/ml). Red, actin; green, E-cadherin; blue, nuclei.

2.3 Video-3, Multilumen cyst (IL-22, 100 ng/ml). Red, ezrin; green, Dlg-1.

2.4 Video-4, Actin protrusions reaching from the basolateral membrane into the matrigel. Red, actin; green, E-cadherin; blue, nuclei.

2.5 Video-5, Detail of the actin protrusions demonstrated in video #4. Red, actin; green, E-cadherin; blue, nuclei.

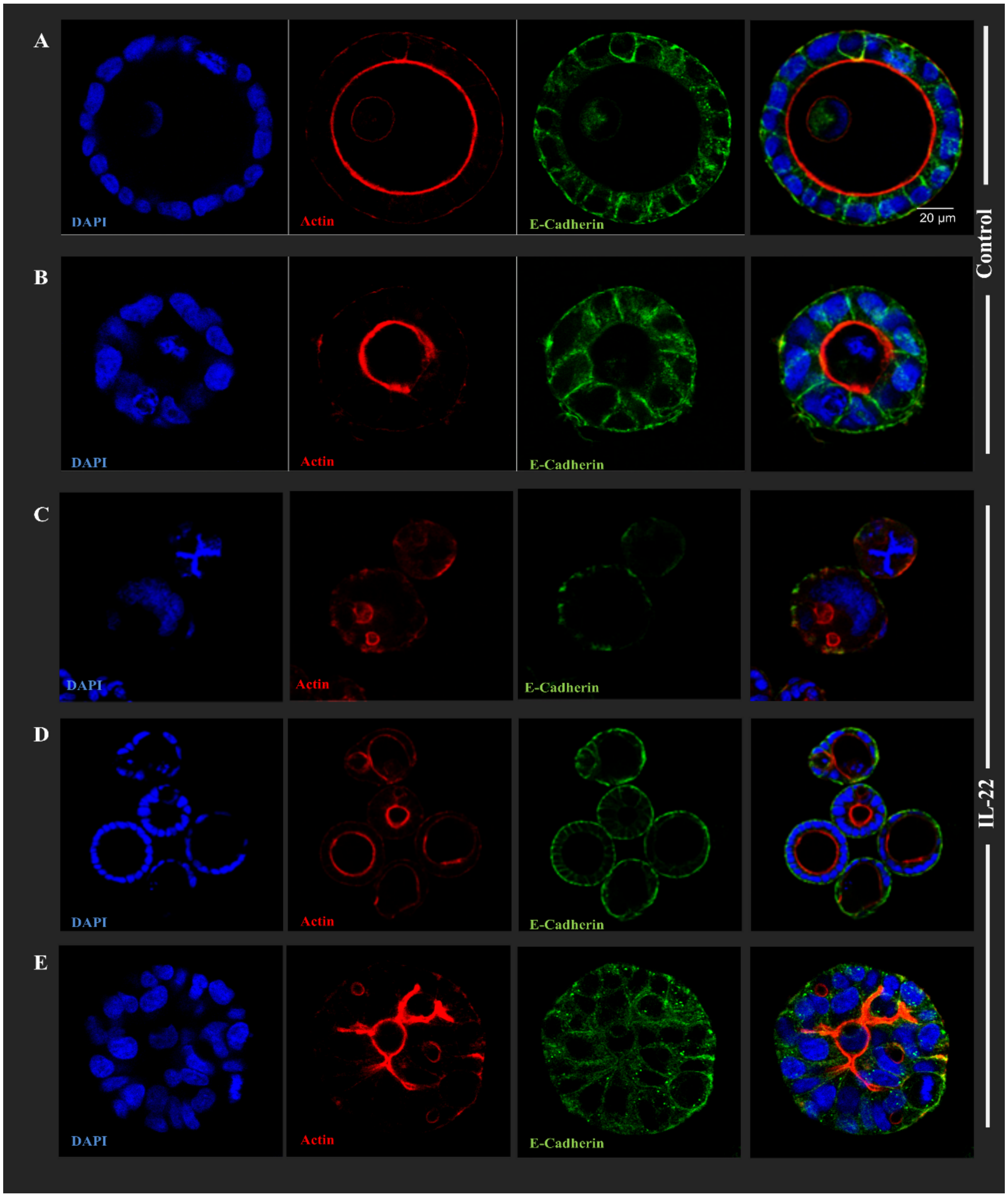

Supplementary Figure 1:
Confocal microscopy of different features observed in cysts grown in Matrigel®. Red: Actin (phalloidin staining), green: E-Cadherin, blue: nuclei. **(A)** Hollow, regular cyst with monolayer, luminal actin staining and basolateral E-cadherin staining. Note the intraluminar nuclear (DAPI-) staining, pointing to lumen formation by intraluminar apoptosis. B: Monolayer cyst with actin and E-cadherin containing protrusions. Note the interrupted luminal actin lining on the surface of the same cell forming basal blunt actin protrusions. **(C)** Spheres with atypical mitosis and atypical actin expression pattern. **(D)** Assembly of cysts with regular single lumen or multi-lumen. Note that enhanced E-cadherin staining in cysts forming multiple lumina is not restricted to the basolateral membrane. **(E)** Complex cyst, with multiple connected lumina. Note the enhanced luminal staining for actin.

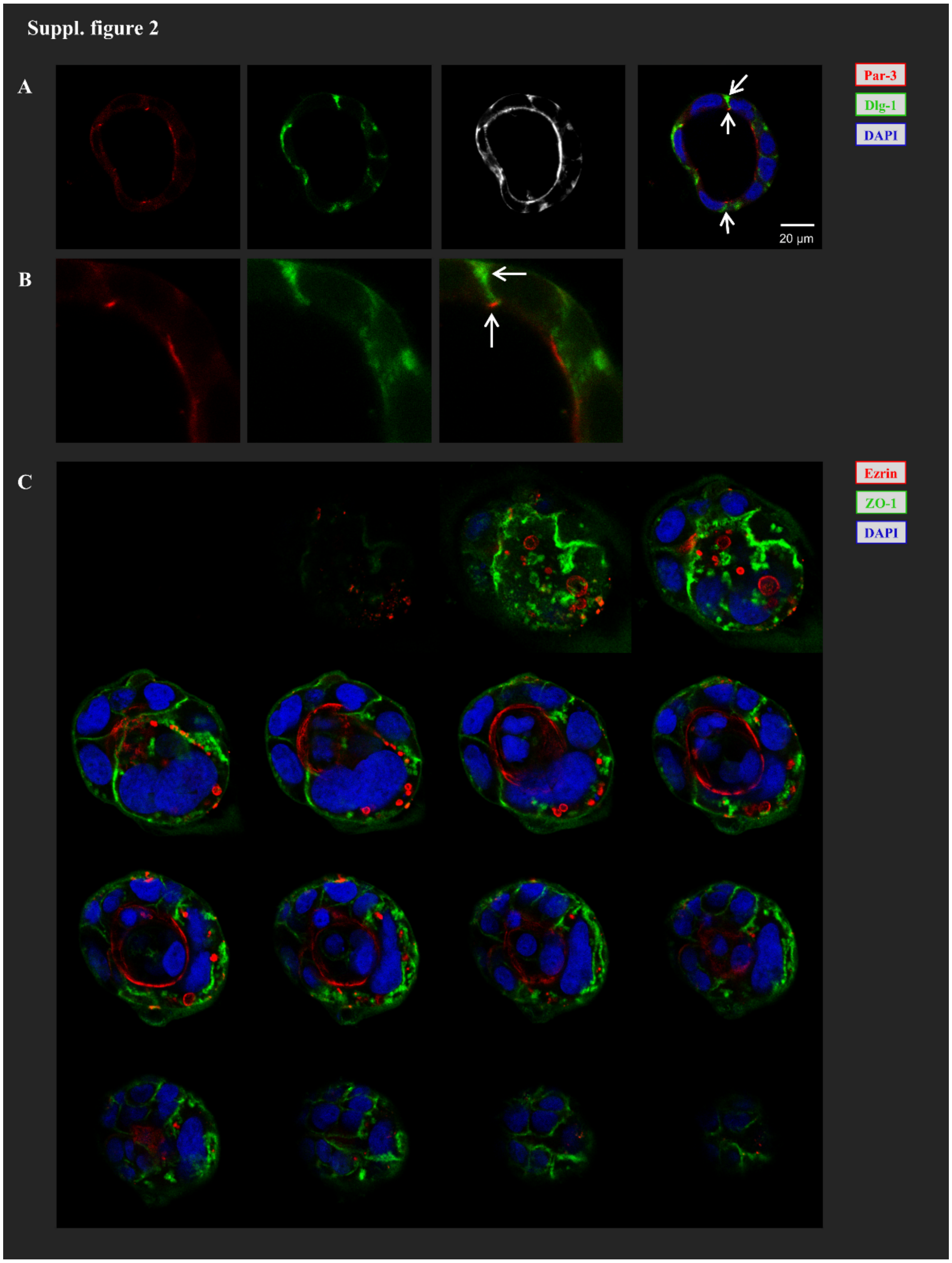

Supplementary Figure 2:
(A) Confocal LSM image of an immunostained 3dimensional CaCo-2 cyst growing in Matrigel. Par-3 (red) is located to the apical part of the lateral membrane (TJ staining) and is distinct from Dlg-1 staining (green). Nuclear staining is blue. **(B)** Detail of (A) revealing the non-overlapping Par-3 and Dlg-1 stainings. **(C)** Gallery display of a multicolor Z-stack revealing multilumen formation in a 3D CaCo-2 cyst after treatment with IL-22 (10 ng/ml).

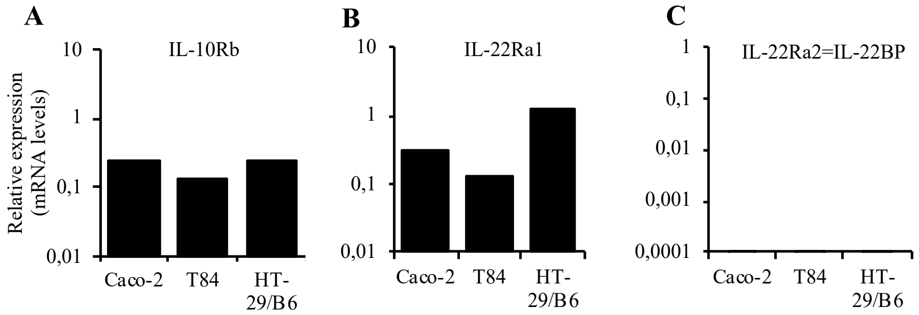

Supplementary Figure 3:
RNA levels of the IL-22 receptor subunits, i.e. IL10Rb **(A)** and IL-22Ra1 **(B)**, as well as the IL-22 binding protein (IL-22BP, **C**) as determined by RT-qPCR in the three intestinal epithelial cell lines that were used in the present study (CaCo-2, T84, HT-29/B6).

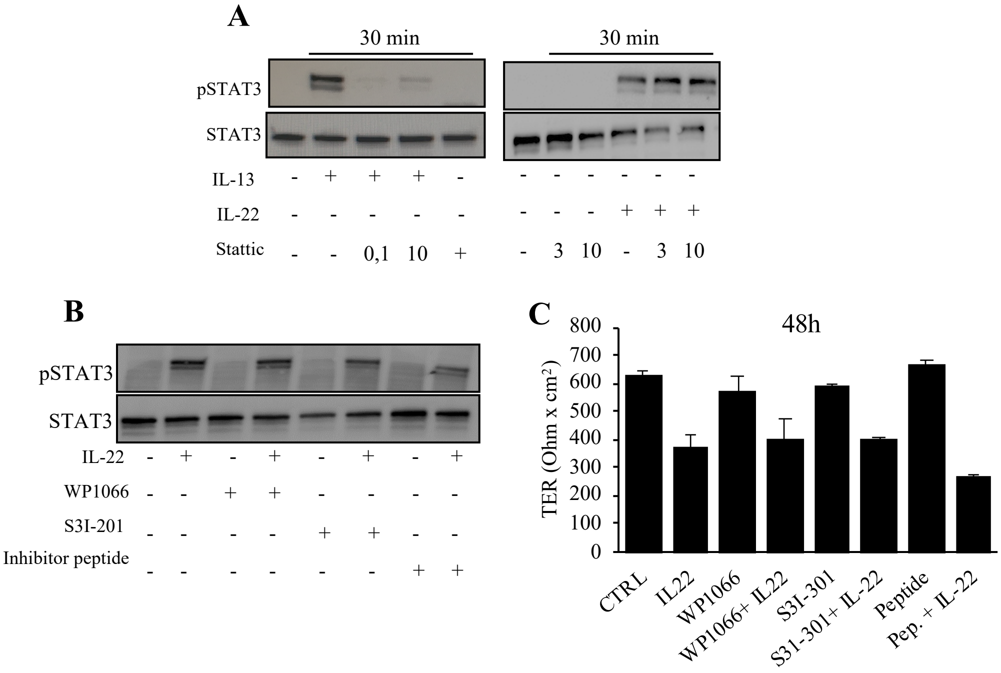

Supplementary Figure 4:

Additional STAT3 inhibitor studies. HT-29/B6 cells were treated with (A) IL-22 (10 ng/ml) or IL-13 (10 ng/ml) and the (B) STAT3 inhibitors Stattic, WP1066, S3I-201 and the inhibitor peptide as described in the Methods section (C) TER of the filter treated with STAT3 inhibitors with or without IL-22 (10 ng/ml) exposure was measured after 48 hours. n=16 filters. While inhibition of IL-13-mediated STAT3 activation was accomplished easily, IL-22-mediated STAT3 activation was resistant to most STAT3 inhibitors.
