# Supplementary material for: Reprogramming Intestinal Epithelial Cell Polarity by Interleukin-22
Source: Front Med (Lausanne). 2021 Apr 12;8:656047. doi: 10.3389/fmed.2021.656047 (PMC8072225; doi:10.3389/fmed.2021.656047)
Supplement: Supplementary file 2 [file Table_1.docx]

| Supplementary table 1: STAT3 inhibitors | | | | |
| --- | --- | --- | --- | --- |
|  | **Name of inhibitor** | **Manufacturer** | **Concentration (µM)** | **Catalog #** |
| STAT3 inhibitors | Stattic | Calbiochem  (San Diego, USA) | 0.1; 3; 10 | 573099 |
|  | WP1066 | Calbiochem  (San Diego, USA) | 10; 50 | 573097 |
|  | STAT3 Inhibitor VI  (S3I-201) | Calbiochem  (San Diego, USA) | 100 | 573192 |
|  | Cell permeable  STAT3 Inhibitor Peptide | Calbiochem  (San Diego, USA) | 100 | 573096 |
|  |  |  |  |  |
| MAPK/ERK inhibitor | U0126 | Biogems International  (Westlake Village, USA) | 10 | 1095821 |

| Supplementary table 2: Primary antibodies for western blotting | | | | |
| --- | --- | --- | --- | --- |
| Antigen | **Dilution** | **Host animal** | **Manufacturer** | **Catalog #** |
| AKT | 1:1000 | Rabbit | Cell Signaling Technology  (Frankfurt am Main, Germany) | 9272S |
| pAKT  (Thr308) | 1:1000 | Rabbit | Cell Signaling Technology  (Frankfurt am Main, Germany) | 9275S |
| ERK | 1:1000 | Rabbit | Cell Signaling Technology  (Frankfurt am Main, Germany) | 4695S |
| pERK | 1:1000 | Rabbit | Cell Signaling Technology  (Frankfurt am Main, Germany) | 91015S |
| STAT3 | 1:1000 | Rabbit | Cell Signaling Technology  (Frankfurt am Main, Germany) | 30835S |
| pSTAT3  (Tyr705) | 1:1000 | Rabbit | Cell Signaling Technology  (Frankfurt am Main, Germany) | 9145S |
| Claudin 1 | 1:1000 | Rabbit | Thermo Fisher Scientific (Massachusetts, USA) | 51-9000 |
| Claudin 2 | 1:1000 | Mouse | Thermo Fisher Scientific (Massachusetts, USA)) | 516100 |
| Claudin 4 | 1:1000 | Mouse | Thermo Fisher Scientific (Massachusetts, USA) | 329400 |
| E-cadherin | 1:1000 | Mouse | Cell Signaling Technology  (Frankfurt am Main, Germany) | 14472S |
| JAM-A | 1:1000 | Rabbit | Thermo Fisher Scientific (Massachusetts, USA) | 36-1700 |
| β-actin | 1:1000 | Mouse | Sigma Aldrich  (St. Louis, USA) | A5441 |
| MMP-7 | 1:500 | Rabbit | Abcam  (Cambridge, UK) | EPR17888-71 |
| For detection of primary antibodies, peroxidase-conjugated secondary goat anti-rabbit IgG or goat anti-mouse IgG were used from Jackson ImmunoResearch, Ely, UK at a dilution of 1:10,000. | | | | |
